# Implications of Family-Oriented Psychotherapy for Hereditary Angioedema Management: A Case Series

**DOI:** 10.7759/cureus.65526

**Published:** 2024-07-27

**Authors:** Abyson Kalladanthyil, Ami J Patel, Rama R Gogineni

**Affiliations:** 1 Psychiatry, Cooper Medical School of Rowan University, Camden, USA; 2 Internal Medicine, Cooper Medical School of Rowan University, Camden, USA; 3 Psychiatry, Cooper University Hospital, Camden, USA

**Keywords:** stress management, chronic conditions, hereditary angioedema, heritable conditions, family-centered psychotherapy

## Abstract

The role of psychotherapy in the management of chronic conditions has been widely explored and supported. The current approach to the utilization of family-oriented psychotherapy in treatment plans is individualized to the patient and focused on the development of personal coping skills alongside identifying and changing negative thoughts, emotions, and behaviors. Alleviation of symptom burden, improvement in psychiatric co-morbidities like anxiety and depression, and enhancement of quality of life have all been found to be associated with incorporating family-oriented psychotherapy in the management of chronic conditions. In contrast, heritable conditions, such as hereditary angioedema (HAE), have not been the center of extensive research. Heritable conditions introduce a new category of stressors that require management like the anxiety of a parent, a sibling, a child, or another family member decompensating at the same time as oneself. Family-centered psychotherapy focuses on discussing the stressors of the family unit and the development of coping strategies to prevent the time course of one family member’s condition from exacerbating another family member’s condition. This model has been utilized for families with separate chronic conditions, but its role and effectiveness in managing inherited conditions have room for investigation. This paper presents a case series on a family engaging in family-centered psychotherapy for HAE.

## Introduction

Hereditary angioedema (HAE) is a rare autosomal dominant disorder that affects 1/50,000 individuals worldwide [1]. The pathophysiology of this disorder is a deficiency or dysfunction of the C1 esterase inhibitor (C1-INH), which is a key component of the complement, coagulation, fibrinolysis, and kallikrein-kinin systems [1]. If C1-INH is deficient or dysfunctional, there is continuous production of kallikrein, which is responsible for cleaving high molecular weight kininogen into bradykinin [1]. Continuous production of kallikrein will ultimately lead to overproduction and accumulation of bradykinin. Bradykinin increases vascular permeability, leading to angioedema [1].

There are three subtypes of HAE, which are all bradykinin-mediated. Type I is a quantitative deficiency of C1-INH that accounts for about 85% of cases while type II is a qualitative dysfunction of C1-INH that accounts for the rest of the cases [1]. Type III is estrogen-dependent with normal C1-INH activity, but several specific gene mutations have been found to be associated. Due to the estrogen dependence of type III, affected individuals are predominantly females, and the presentation of symptoms appears later in life [2]. Type I and type II show presentations more commonly in the second decade of life, and young children are typically asymptomatic [2]. The symptomatic presentation varies from individual to individual, but the most classical is non-pitting angioedema and abdominal pain [3]. The angioedema most frequently involves the face, and laryngeal edema is a major complication of an acute attack [3,4]. Laryngeal edema can lead to air compromise and asphyxiation [4]. The gastrointestinal tract can also be affected, which can result in debilitating visceral abdominal pain [4]. Acute episodes may have a preceding trigger for some individuals such as trauma, medical procedures, stress, infections, and starting angiotensin-converting enzyme inhibitor therapy; however many individuals have no consistent identifiable triggers [4]. 

The multitude of triggers and burdens of this illness causes patients with HAE to have an increased risk of mental health disorders [5]. Tunçel et al. found that HAE significantly impairs quality of life, which correlates with depression, anxiety, perceived social support, and perceived limitations. They also found that higher levels of C1-inhibitor function and C1q, the first subcomponent of the C1 complex of the classical pathway of complement activation, correlate with better quality of life. They also propose that patients with HAE who suffer from depressive and anxious symptoms be referred to psychiatrists as this can further decrease quality of life and augment perceived discrimination [6]. In children with HAE, the incidence of alexithymia or impaired regulation and processing of emotions has been found to be higher. This in itself compounds the baseline stress, anxiety, and low mood the patient may be experiencing, ultimately becoming a trigger of edema attacks [7].

In this case series, we report a family who has been suffering from HAE and will discuss the intergenerational mental health implications this condition has within one nuclear family as well as the role family-centered psychotherapy has had in the management of their disease.

## Case presentation

This family of four integrated by a married couple with a son and daughter was initially referred to the child psychiatrist due to the son’s suicidal ideation, at which point his sister and mother were encouraged to participate in the family-centered psychotherapy as well. The father of the children, while not afflicted with HAE, also actively participates in psychotherapy and is a key supportive figure for the family. Specific focus was placed on positive psychotherapy to develop coping skills for the family unit.

Case one: mother

A 57-year-old female began experiencing HAE flares in childhood. Her records from that time could not be located for the exact age of her initial flare. Her most recent flare was in 2022 due to uncontrolled pain from lumbar radiculopathy. She has been seen in the psychiatric outpatient setting since 2015 for post-traumatic stress disorder (PTSD) and generalized anxiety disorder (GAD), which were diagnosed shortly after her first HAE flare. Her two children have also inherited HAE and have also been diagnosed with GAD and major depressive disorder (MDD) with psychotic features secondary to their known, unpredictable medical condition, all of which have exacerbated her personal anxiety. 

Her psychiatric history is additionally significant for separation anxiety and alexithymia as the patient's daughter reports the patient suppresses many of her thoughts and emotions to avoid worrying the family. In times when her kids have been suffering from HAE flares and ridiculed by others, causing them strife, she endorses feelings of helplessness. 

Her current psychiatric management comprises consistent family-oriented psychotherapy and medication management including olanzapine, oxcarbazepine, lorazepam, haloperidol, and sertraline all at adequate dosages. She attends psychotherapy sessions with her two children, which focus on improving her ability to communicate her emotions and thoughts with her children. Family-oriented psychotherapy was first initiated on a weekly basis and then transitioned to monthly sessions once symptoms were effectively managed.

Case two: daughter

A 29-year-old female began receiving treatment for HAE in 2010 after experiencing an upper respiratory infection-induced flare. Her medical treatment consists of prophylactic intravenous infusions of C1-INH concentrate at therapeutic doses. The following year, she experienced flares every few months, which led to increased absenteeism from school and ultimately decreased academic performance. 

Her psychiatric history is significant for ADHD, which was diagnosed in childhood, GAD, and PTSD, which was diagnosed after her first HAE flare in 2010. Her last documented HAE flare was in 2011. She was started on escitalopram at that time and titrated to a therapeutic dose. Her anxiety would lead to stress-induced binge eating that resulted in substantial weight gain. The increased stress from her poor academic performance compounded with her anxiety of experiencing another HAE flare led to increased fatigue, sleepiness, appetite, and weight gain. She was subsequently diagnosed with MDD, and her medication regimen was changed to sertraline 100 mg. As she began her college career, she endorsed her PTSD, ADHD, anxiety, and depression all significantly interfered with day-to-day functioning and school performance. 

Her current psychiatric management comprises psychotherapy, which was started when her brother had a suicide attempt in 2016. She has been engaging in family-oriented psychotherapy with her brother and mother since 2016 along with pharmacologic interventions. The frequency of family-oriented psychotherapy, just as with the mother, was first initiated on a weekly basis and then transitioned to monthly sessions once symptoms were effectively managed. During her initial evaluations and therapy sessions, she was noted to maintain a positive image and not communicate the post-traumatic effects of her and her family’s struggle with HAE. Long-term consistent psychotherapy has focused on building stress reduction strategies to aid in managing her psychiatric well-being and also prevent stress-induced HAE flares. 

Case three: son

A 27-year-old male began receiving treatment for HAE in 2009. His medical treatment consists of prophylactic intravenous infusions of C1-INH concentrate at therapeutic doses. He experienced two hospitalizations by February 2011 for abdominal pain secondary to a HAE flare. He has had significant school absenteeism, multiple emergency room visits for HAE flares, and inadequate pain management with opioids. He has a medical history significant for obesity that was exacerbated by systemic corticosteroids that were required pre-infusions for asthma. 

His psychiatric history is significant for GAD, MDD, PTSD, attention deficit hyperactivity disorder, self-harm behaviors, and suicidal ideation and multiple attempts, for which he has received outpatient psychiatric treatment since 2015. His psychiatric conditions are related to and frequently exacerbated by his frustrations and stress in regard to caring for his mother and sister as well as dealing with his HAE flares and long-term treatment requirements. 

His current psychiatric management is consistent psychotherapy to enhance his functionality, mood stabilization, and development of coping strategies to navigate his and his family’s labile, chronic conditions coupled with pharmacological therapy including dextroamphetamine-amphetamine, sertraline, gabapentin, and lorazepam at adequate dosages. His last angioedema flare that required emergency medicine evaluation was in November 2014. The documentation from that visit states he presented with severe abdominal pain, and edema was anxious, and endorsed significant school-related stress.

Outcomes

All three family members are stable and have been able to continue keeping their HAE exacerbation under control with the help of their medication management and regular family-oriented psychotherapy sessions. They have continued to lead productive lives even when negative life circumstances continue to present themselves. They have developed coping strategies with the help of their psychiatrist and have been able to ensure that the stresses they do not have control over are able to be managed. They report that the open communication developed from family-oriented psychotherapy has significantly alleviated anxiety and stress related to each other and equipped them with coping strategies to focus on their personal mental health. For example, the husband of the mother (father of the children) developed a terminal illness that has been a significant point of stress for this family. Through this challenging circumstance, they have continued to effectively communicate their emotions during the psychotherapy sessions led by their psychiatrist to prevent stress-induced HAE flares. Both children have additionally been able to finish their education and maintain employment with the coping strategies that were developed through the psychotherapy sessions.

## Discussion

Internal and external psychological stressors can be triggers to HAE. Stress is actually the most frequently cited trigger [8]. The unpredictability of HAE also leads to increased levels of stress, leaving patients in a particularly curious circumstance. During the COVID-19 pandemic, one of the most recent global stressors, it was found that disease morbidity and psychological stress outcomes related to HAE worsened and common co-morbid diagnoses of anxiety, depression, and PTSD were associated with worse outcomes [9]. As patients suffer from HAE attacks, they accumulate anxiety and stress, which then leave them more prone to another attack; they live in a self-fulfilling prophecy. Management of stress utilizing psychotherapy can be integrated to better manage these symptoms in hopes of curbing upcoming attacks.

The benefits of psychotherapy for chronic conditions such as asthma, diabetes, cancer, and chronic pain have been widely studied and supported. Cognitive behavior therapy (CBT), including newer forms such as acceptance and commitment therapy, has shown significant effectiveness in managing chronic pain by improving the patient’s ability to cope with pain, engage in activities of daily living, and overall quality of life [10]. More broadly, CBT has been shown to aid in psychological adjustment to a diagnosis of a chronic medical condition and reduce the symptom burden of common psychological co-morbidities to chronic conditions like anxiety and depression [11]. The framework for the effectiveness of psychotherapy focuses on the connections between thoughts, emotions, behaviors, and physical symptoms [12]. Negative thoughts or ruminations about a chronic condition can lead to emotions of anxiety and depression. These emotions can then encourage hypervigilance and/or avoidance behaviors [12]. The behaviors can then manifest in physical symptoms such as increased heart rate or shortness of breath, which will ultimately validate the precipitating thoughts [12]. CBT emphasizes focusing on the present and being goal-oriented, which facilitates dissociation of the thoughts, emotions, behaviors, and physical symptoms cycle at the thoughts and behaviors stage [12]. A vast majority of the literature on the role of psychotherapy in the management of chronic conditions focuses on an individualized approach and does not adequately represent heritable, chronic conditions.

The implementation of family-centered psychotherapy between all three of the members of this family who all suffer from HAE was not only critical to the management of their own chronic illness, but it stood to make them resilient enough to withstand the stresses of each other’s chronic illness. In contrast to other chronic conditions where family is often viewed as a protective factor and a valuable element of strong social support, family serves as both a protective and precipitating factor for heritable conditions. Positive strategic family and individual psychotherapy enables the identification of interpersonal relationships that comprise a family unit and possible stressors existent in those relationships to then implement coping strategies to mitigate the stress, while still preserving the relationship [13,14]. If one member of this family experiences an HAE flare, the event would subsequently be a new stressor for the whole family unit. Family-centered psychotherapy can encourage familial resilience while simultaneously decreasing stress-induced angioedema flares. Since engaging in family-centered psychotherapy, both children in this case series have not required hospitalization for a stress-induced flare in 10 years. The family unit continues to navigate difficult educational, vocational, physical, mental, and emotional situations. With the coping skills and open communication facilitated by family-centered psychotherapy, they are able to focus and work on improving those aspects of their life without exacerbating their HAE.

## Conclusions

Heritable conditions would benefit from family-oriented early, psychotherapy for the management of individual stress as well as group stress. This case series illustrates how HAE not only affects individuals physically but also places a significant emotional and psychological burden on entire families. Integrating family-centered psychotherapy into the treatment plans for families dealing with HAE has shown promise in enhancing coping skills, improving overall mental health, and stabilizing the emotional dynamics within the family unit. By improving the emotional dynamics within the family unit, there can be a reduction in stress-induced angioedema flares, hospitalizations, and symptom burden of psychiatric co-morbidities. Moving forward, the adoption of family-centered psychotherapy for managing other hereditary conditions may be more effective and rewarding compared to individualized psychotherapy as the implications of family dynamics play a larger role in inherited conditions.
